# Perianal streptococcal disease in childhood: systematic literature review

**DOI:** 10.1007/s00431-021-03965-9

**Published:** 2021-02-02

**Authors:** Renato Gualtieri, Gabriel Bronz, Mario G. Bianchetti, Sebastiano A. G. Lava, Elena Giuliano, Gregorio P. Milani, Luca M. M. Jermini

**Affiliations:** 1 Pediatric Institute of Southern Switzerland, Ospedale San Giovanni, Bellinzona, Switzerland; 2 Family Medicine Institute, Faculty of Biomedical Sciences, Università della Svizzera Italiana, Lugano, Switzerland; 3 Pediatric Cardiology Unit, Department of Pediatrics, Centre Hospitalier Universitaire Vaudois and University of Lausanne, Lausanne, Switzerland; 4 Pediatric Unit, Fondazione IRCCS Ca’ Granda Ospedale Maggiore Policlinico, via della Commenda 9, 20122 Milan, Italy; 5 Department of Clinical Sciences and Community Health, Università degli Studi di Milano, Milan, Italy

**Keywords:** Childhood, Perianitis, Streptococcus, Anal dermatitis, Perianal cellulitis

## Abstract

Group A Streptococcus has been associated with a perianal infection. We conducted a systematic review of the literature on childhood streptococcal perianitis in three databases: Excerpta Medica, National Library of Medicine, and Web of Science. The main purposes were to document the clinical features, the tendency to recur, the association with an asymptomatic streptococcal throat carriage, the accuracy of rapid streptococcal tests, and the mechanism possibly underlying the acquisition of this infection. More than 80% of cases are boys ≤7.0 years of age with defecation disorders, perianal pain, local itch, rectal bleeding, or fissure and a sharply demarcated perianal redness. Perianitis is associated with a streptococcal tonsillopharyngitis in about every fifth case. The time to diagnosis is ≥3 weeks in 65% of cases. Recurrences occur within 3½ months in about 20% of cases. An asymptomatic group A streptococcal throat carriage occurs in 63% of cases. As compared with perianal Streptococcus A culture, the rapid streptococcal tests have a positive predictive value of 80% and a negative predictive value of 96%. It is hypothesized that digital inoculation from nasopharynx to anus underlies perianitis. Many cases are likely caused directly by children, who are throat and nasal carriers of Streptococcus A. Some cases might occur in children, who have their bottoms wiped by caregivers with streptococcal tonsillopharyngitis or carriage of Streptococcus.

*Conclusion*: Perianitis is an infection with a distinctive presentation and a rather long time to diagnosis. There is a need for a wider awareness of this condition among healthcare professionals.

**Table:** 

**What is Known:** • *Group A Streptococcus may cause perianitis in childhood.* *• Systemic antimicrobials (penicillin V, amoxycillin, or cefuroxime) are superior to topical treatment.*	
**What is New:** • *The clinical presentation is distinctive (defecation disorders, perianal pain, local itch, rectal bleeding, or fissure and a sharply demarcated perianal redness).* *• The time to diagnosis is usually ≥3 weeks.* *Recurrences occur in about 20% of cases.*

**What is Known:**

• *Group A Streptococcus may cause perianitis in childhood.*

*• Systemic antimicrobials (penicillin V, amoxycillin, or cefuroxime) are superior to topical treatment.*

**What is New:**

• *The clinical presentation is distinctive (defecation disorders, perianal pain, local itch, rectal bleeding, or fissure and a sharply demarcated perianal redness).*

*• The time to diagnosis is usually ≥3 weeks.* *Recurrences occur in about 20% of cases.*

## Introduction

First reported in 1966, perianal infection associated with group A Streptococcus [1], for simplicity subsequently referred to as perianitis, is a pediatric disease that presents with sharply demarcated redness, mostly associated with local signs of inflammation [2].

A few months ago, we treated a child affected by perianitis [3] and were impressed by the paucity of recent literature on this condition. To efficiently integrate the existing information on perianitis, we conducted a systematic review of the original literature. The main purposes were to document the clinical features (including the occurrence of immunologically mediated sequelae and the association with tonsillopharyngitis), the prevalence, the seasonality, the tendency to recur, the association with an asymptomatic streptococcal throat carriage, the accuracy of rapid streptococcal tests, and, finally, the acquisition and the transmission of the disease.

## Methods

### Search strategy

We followed [4] the 2020 guideline for reporting systematic reviews. Databases searched were Web of Science, Library of Medicine, and Excerpta Medica up to December 2020 without language restriction. Search terms were “peri-anal cellulitis,” “perianal cellulitis,” “peri-anal streptococcal cellulitis,” “perianal streptococcal cellulitis,” “peri-anal dermatitis,” “perianal dermatitis,” “peri-anal streptococcal dermatitis,” “perianal streptococcal dermatitis,” “peri-anal streptococcal disease,” “perianal streptococcal disease,” “peri-anal streptococcal infection,” “perianal streptococcal infection,” “streptococcal anitis,” “streptococcal peri-anitis,” “streptococcal perianitis”. References listed within bibliographies of the retrieved records and personal files of the authors were also considered for inclusion.

Two authors independently screened all identified titles and abstracts in a nonblinded fashion. Upon recovery of candidate reports, full-text publications were reviewed for eligibility. During the entire process, uncertainties were resolved through team discussions and consensus.

### Eligibility criteria

We searched original reports published after 1965 that documented patients affected by a perianal streptococcal disease. For the purpose of this study, we included individually documented cases of perianitis in subjects 18 years or less of age with a sharply demarcated redness extending 2–4 cm around the anus (often with local signs of inflammation such as superficial edema, infiltration, and tenderness), accompanied by a positive perianal bacteriological culture for group A Streptococcus. Cases with clinical features consistent with the diagnosis of streptococcal perianal infection but without a bacteriological culture were excluded. Cases supported uniquely by a positive rapid streptococcal test were also not included. For each case, we recorded the following: demographics, symptoms, and findings including fever, anal pain, anal itch, defecation disorders, rectal bleeding, anal fissures, discharge, and associated vaginal, penile, or cutaneous (impetigo, scarlatiniform rash) involvement, time to diagnosis, i.e., duration of symptoms prior to diagnosis and bacteriological studies. The possible occurrence of immunologically mediated sequelae such as acute rheumatic fever, acute glomerulonephritis, erythema nodosum, pediatric autoimmune neuropsychiatric disorder associated with group A streptococci, poststreptococcal myalgia, and psoriasis was also addressed. The accurateness in describing symptoms, physical findings, and of sequelae was used to grade the completeness of reporting as high or or low.

Reports analyzing the prevalence of the condition, its seasonality, the tendency to recur, the association with a streptococcal tonsillopharyngitis or an asymptomatic streptococcal throat carriage, the accuracy of rapid streptococcal tests, and the occurrence of community and familial outbreaks were also included and analyzed.

### Analysis - statistics

Categorical variables are presented as frequency (and percentage), continuous variables as median and interquartile range. The two-tailed Mann-Whitney-Wilcoxon test was used for statistics. *P* values <0.05 were considered significant.

## Results

### Search results

**Fig. 1:**
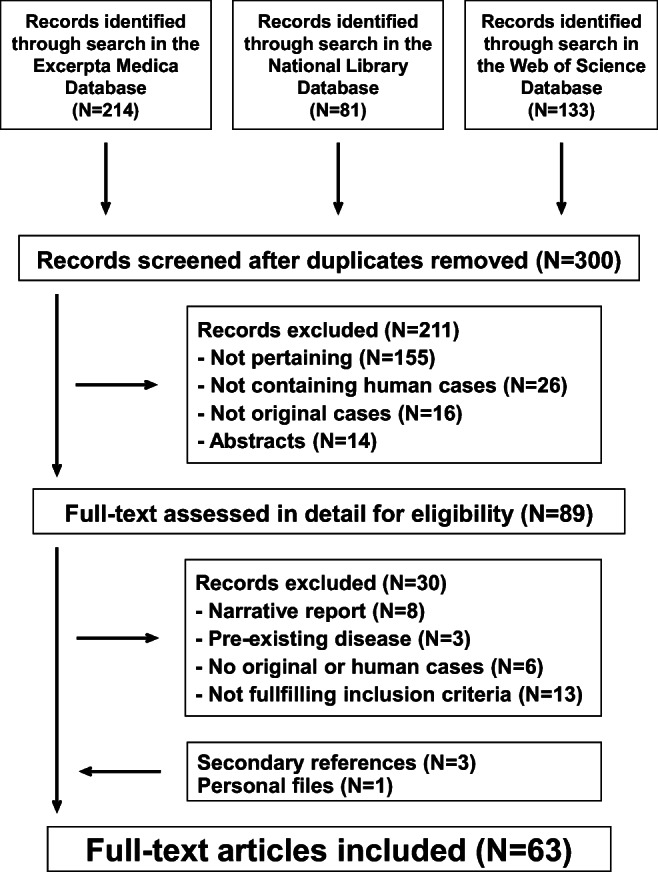
Perianal streptococcal disease in childhood. Flowchart of the literature search process

The literature search process is summarized in Fig. 1. For the final analysis, we retained 63 reports [1, 3, 5–65] published after 1965: 40 from Europe, 18 from North America, three from Asia, and two from Australia. Forty-five reports were published in English, nine in Spanish, three each in French and German, two in Italian, and one in Portuguese.

### Findings

#### Demographics, clinical features, association with a tonsillopharyngitis

We found 47 articles [3, 6–17, 19, 21–23, 25–29, 31–36, 38, 39, 41, 44–52, 54, 58, 61–64] describing 147 individually documented children affected by perianitis, as shown in Table 1. Of note, more than 80% were male and 1.1 to 7.0 years of age. Boys (4.0 [3.0–5.1] years) and girls (4.0 [1.9–5.2] years) did not significantly differ for age. Furthermore, the time to diagnosis was ≥3 weeks in 65% of children. In addition to the sharply demarcated redness around the anus, the main presenting symptoms and signs were, in decreasing order of frequency, defecation disorders, perianal pain, local itch, rectal bleeding, fissure, and fever (Table 1). A concurrent genital involvement (balanitis, *N* = 11; vulvovaginitis, *N* = 3) was noted in slightly more than 10% of cases. Seventeen (11%) immunologically mediated sequelae were detected in the 147 children: 16 cases of psoriasis and one case of poststreptococcal myalgia. No further immunologically mediated sequelae were observed. The accurateness in reporting the cases was high in 102 and low in 45 cases.

**Table 1:** Demographics, history, and clinical features in 147 children 0.1 to 13 years of age affected by streptococcal perianitis. Continuous data are presented as median and interquartile range, categorical data as frequency and percentage

Demographics	
Gender (males : female)	119 (81%) : 28 (19%)
Age	
Years	4.0 [3.0–5.1]
≤1.0 years	15 (10%)
1.1–7.0 years	118 (80%)
≥7.1 years	14 (9.5%)
Time to diagnosis	
Weeks	5 [1–6]
≤2 weeks	51 (35%)
3–5 weeks	49 (34%)
≥6–10 weeks	46 (31%)
Presenting symptoms	
Perianal pain	68 (46%)
Anal itch	67 (45%)
Defecation disorders*	58 (39%)
Rectal bleeding	27 (18%)
Anal fissure	23 (15%)
Discharge	16 (10%)
Fever	7 (4.7%)
Extended skin involvement	
Genital^❖^	16 (11%)
Impetigo	3 (2%)
Scarlatiniform rash	1 (0.6%)
Immunologically mediated sequelae	
Psoriasis^◆^	16 (11%)
Poststreptococcal myalgia	1 (0.6%)
Further sequelae	0 (0.0%)

*Painful defecation (*N* = 44), constipation (*N* = 10), or fecal incontinence (*N* = 4)

^❖^Balanitis (*N* = 13), vulvovaginitis (*N* = 3)

^◆^Guttate psoriasis (*N* = 15), plaque psoriasis (*N* = 1)

The reports detailing the 147 children do not unambiguously address the possible concurrent occurrence of perianitis and tonsillopharyngitis. The latter issue was investigated in two case series [33, 56] including 62 children affected by perianitis. The mentioned reports found that 12 (19%) patients presented simultaneously the clinical picture of a perianitis and a Streptococcus-positive tonsillopharyngitis.

#### Prevalence

Seven retrospective chart reviews addressed the infection rate. Six articles, five from the USA [1, 3, 8, 33, 56] and one from Spain [65], provided data allowing the estimation of the perianitis rate per 10,000 patient encounters in a primary pediatric care setting. The mentioned value was found to be highly variable: from 4.5 to 50, median 16 cases per 10,000 encounters (Table 2). The infection rate was relevantly higher, 480 per 10,000 encounters, in a Swiss University Pediatric Emergency Department mostly caring for referred patients [42].

**Table 2:** Rate (cases per 10,000 patient encounters) of perianal streptococcal disease in general pediatric practice reported in the literature

Author	Country	Period	Cases (*N*)	Cases (per 10,000 encounters)
Amren [1]	USA	May 1964–Jul. 1965	10	4.6
Kokx [8]	USA	Oct. 1985–Jun. 1986	31	46
Combs [30]	USA	Jan. 1990–Mar. 1990	2	16
Mogielnicki [33]	USA	Jan. 1997–Dec. 1997	23	50
Clegg [56]	USA	Jul. 1999–Jun. 2002	56	4.4
Clegg [56]	USA	Jan. 2007–Dec. 2012	101	4.8
Martínez Blanco [65]	Sain	Apr. 2011–Mar. 2019	95	33

#### Seasonality

Eight cases series including a total of 725 cases (from 19 to 157, median 105 cases per communication) investigated the seasonality of perianitis in countries with a temperate climate and found that the condition is most common during cold months [33, 43, 53, 55–57, 59, 65].

#### Recurrences

Three case series including 314 cases [53, 56, 65] found 60 (19%) recurrences 6 months or less after a first episode of perianitis. About 90% of recurrences occurred within 3½ months [53]. Recurrences were also observed in 12 (38%) out of the 31 cases published by Kokx [8]. In the latter report, however, the time to recurrence was not specified.

#### Bacteriological studies

##### Association with an asymptomatic group A streptococcal throat carriage

The throat culture test for A Streptococcus was performed in 66 children with perianitis [24, 33, 56] not associated with the clinical features of tonsillopharyngitis and was found to be positive in 41 (63%) of them.

##### Accuracy of rapid streptococcal perianal swab

The accuracy of rapid streptococcal tests was evaluated in three studies [43, 56, 57], which included 247 children with perianitis. As compared with the ordinary perianal Streptococcus A culture, the rapid streptococcal test was found to have a positive predictive value of 80% and a negative predictive value of 96%.

#### Community and family outbreaks

In a rural Danish community, a cluster of perianitis was observed that was centered around a kindergarten. It concurrently affected 12 children (11 males and 1 female) aged from 3 to 12 years. All cases were caused by a single clone of group A Streptococcus. It was assumed that the source of the outbreak was a mother working in the kindergarten, who was an asymptomatic pharyngeal carrier of the mentioned Streptococcus [40].

**Fig. 2:**
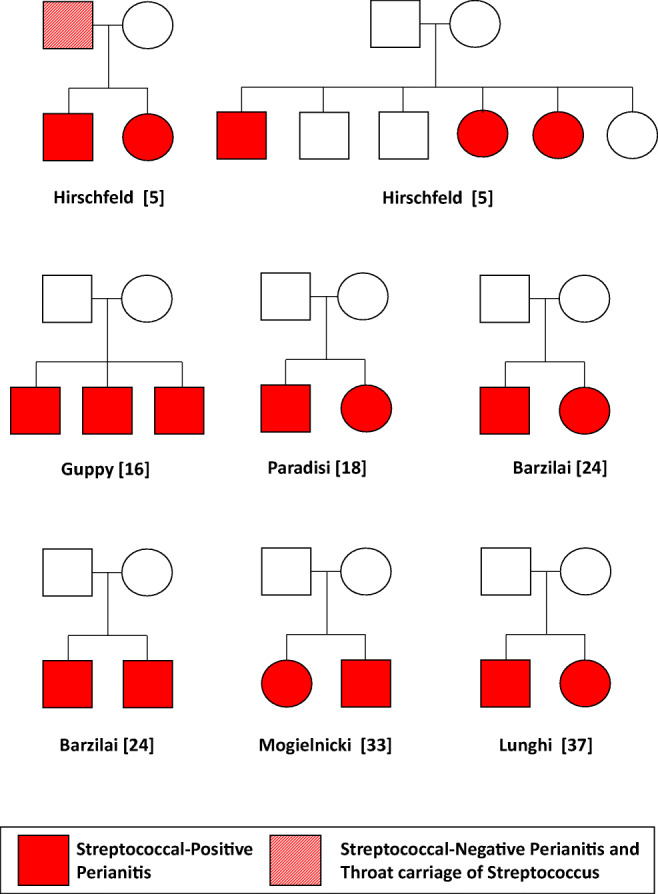
Familial cases of perianal streptococcal disease. Female (circle) and male (square) subjects are given different symbols

We also found 8 families with at least 2 siblings (3 siblings in two families; 2 siblings in 6 families) concurrently affected (Fig. 2) by a streptococcal perianitis (no information on the number of unaffected siblings was available in seven families). Interestingly, a recent history of Streptococcus-positive tonsillopharyngitis and symptoms of perianitis with a negative streptococcal perianal swab were reported in one of the fathers [5, 16, 18, 24, 33, 37].

## Discussion

Perianitis has also been referred to, in decreasing order of frequency, as perianal streptococcal dermatitis, perianal streptococcal cellulitis, and perianal streptococcal infection. The results of this systematic review of the literature may be recapitulated and discussed as follows.

Perianitis presents without signs consistent with a systemic illness. The local features include a sharply demarcated perianal redness accompanied by signs of local inflammation such as superficial edema, infiltration, and tenderness. Further features include defecation disorders, perianal pain, local itch, rectal bleeding, and fissure. Concomitant balanitis or vulvovaginitis occurs in about 10% of cases. Finally, perianitis is associated with a tonsillopharyngitis in about every fifth case.

Although perianitis may occur in adults [2], it is predominantly a disease of childhood. Pediatric cases occur before puberty, with ages ranging from infancy to preteen years and a strong predilection for males. We do not have an explanation for the latter observation. The age distribution of perianitis mirrors that of streptococcal impetigo but markedly differs from that of streptococcal tonsillopharyngitis, which is more common in children 5 to 15 years of age [66]. Like tonsillopharyngitis, perianitis peaks during winter months with frequent recurrences in countries with a temperate climate [66].

The true incidence of perianitis is currently unknown. Nonetheless, this analysis documents the relatively common occurrence of this condition in general pediatric practice and in pediatric emergency medicine.

Perianitis may trigger poststreptococcal myalgia and especially psoriasis, two recognized sequelae of group A streptococcal infections [67, 68]. No further immunologically mediated sequelae were observed.

Rapid streptococcal tests were found to have a positive predictive value of 80% and a negative predictive value of 96%. These figures are almost identical to those already reported for streptococcal tonsillopharyngitis [69].

In healthy subjects [70], throat carriage of A Streptococcus is frequent (especially in winter months). In contrast, anal, vaginal, or penile carriage is very unusual [71, 72]. It is therefore tempting to speculate that Streptococcus swallowing and especially digital inoculation from the nasopharynx to the anal region underlie perianitis. Many cases of inoculation are likely caused directly by children, who are throat and nasal carriers of Streptococcus A. Preschoolers have their bottoms wiped by caregivers. Caregivers affected by streptococcal tonsillopharyngitis or carriers of A Streptococcus (and who usually wash their hands after but not before wiping) have therefore been imputed as a possible cause of inoculation [73].

The hypothesis of inoculation from the nasopharynx is further supported by the following data: (i) the seasonal distribution of perianitis and streptococcal tonsillopharyngitis is similar; (ii) perianitis is associated with a streptococcal tonsillopharyngitis in every fifth case; (iii) the throat test for A Streptococcus is positive in the majority of children with perianitis; (iv) the perianal carrier rate of A streptococci is very low, as previously stated, in healthy subjects [71, 72], but is 6% in children with streptococcal tonsillopharyngitis [74]. The literature also supports the notion of infection spread within families or in daycare centers.

This review did not specifically address the treatment of perianitis. Topical antimicrobial monotherapy, which has been advocated, seems poorly effective, likely because perianal dermatitis affects the deeper layers of the skin [2, 75]. Consequently, most authorities [2] suggest a systemic treatment with either penicillin V or amoxycillin (with or without additional topical therapy). More recently, a small randomized study demonstrated that a 7-day treatment with cefuroxime, a β-lactamase-resistant cephalosporin, might be superior to a 10-day penicillin treatment [75]. The rationale underlying the advantage of cefuroxime might be the co-pathogenicity of β-lactamase producing bacterial strains [75].

Perianal streptococcal disease is sometimes associated with a balanitis or a vulvovaginitis. On the other hand, Streptococcus A has also been associated with a balanitis or a vulvovaginitis in the absence of a perianitis. It has been therefore suggested to summarize perianitis, balanitis, and vulvovaginitis caused by A Streptococcus under the inclusive term perineal streptococcal disease [2].

The most relevant limitation of this analysis comes from the small number of published articles on children affected by perianitis, which were published over more than 50 years. Finally, completeness in reporting cases was sometimes low.

## Conclusion

Although perianitis is an infection with a distinctive presentation, it is often initially confused with conditions such as irritant or allergic dermatitis, pinworm infestation, and child abuse. Misdiagnosing the condition may result in redundant investigations and unnecessary management, which can cause apprehension among caregivers and health professionals. There is a need for a wider awareness of this condition.
